# Stratum corneum lipid liposomes for investigating skin penetration enhancer effects

**DOI:** 10.1039/c8ra04129f

**Published:** 2018-08-02

**Authors:** Mónika Bakonyi, Attila Gácsi, Szilvia Berkó, Anita Kovács, Erzsébet Csányi

**Affiliations:** Institute of Pharmaceutical Technology and Regulatory Affairs, University of Szeged Szeged H-6720 Hungary csanyi@pharm.u-szeged.hu +36-62-545-573

## Abstract

Knowledge of the mechanism of action of skin penetration enhancers is essential to formulators for optimizing formulations and to maximize the efficacy of enhancers. To obtain information about the effects of penetration enhancers as a fast initial screening, investigations have been performed to identify possible correlations of the biological effectiveness of penetration enhancers with their interaction with a well-defined model system consisting of skin mimic lipid bilayers, as determined by calcein release experiments using stratum corneum lipid liposomes (SCLLs). We aimed to investigate the enhancing effects of different concentrations of two chemical penetration enhancers, Kolliphor RH40 and Transcutol on SCLLs. The results obtained by SCLL-based techniques were compared with conventional *ex vivo* penetration studies in case of Kolliphor RH40 to evaluate the potential of SCLLs as an alternative tool for screening various types and concentrations of penetration enhancers. As a result, calcein leakage assay performed with SCLL was considered to be a good model for the skin penetration enhancing effect. This method could be used as a time-saving and sensitive alternative *in vitro* screening technique in the early stage of the development of dermal formulations.

## Abstract 2

The aim of this work was to investigate the applicability of stratum corneum lipid liposomes as *in vitro* skin models for studying skin penetration enhancer effect of Kolliphor RH40 and Transcutol.

## Graphical abstract

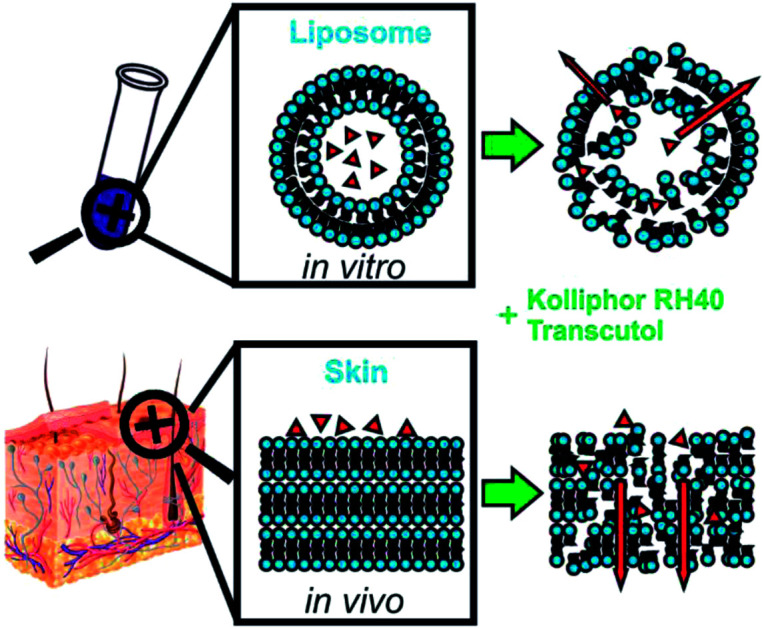

## Introduction

The skin has been recognized as a highly attractive application site of therapeutic agents with both local and systemic effects.^1^ However, the outermost layer of the skin, the stratum corneum (SC) forms a barrier, which makes it hard for active pharmaceutical ingredients (API) to pass through this layer and exert their pharmacological effects.^2^ It has been shown that the transdermal flux of API and SC lipid conformation is strongly correlated.^3^ These lipids are arranged in a bilayer structure; thus, liposomes can be suitable systems to mimic this arrangement *in vitro*.^2^

Stratum corneum lipid liposomes (SCLLs) are composed of a lipid mixture approximating the composition of the SC: ceramides (40%), cholesterol (25%), cholesteryl sulfate (10%) and free fatty acids (25%). This lipid composition is capable of forming unilamellar bilayers at physiological pH, and they were shown to be really stable.^4,5^ These systems can be applied as *in vitro* models for screening agents of pharmaceutical or cosmetic interest, enabling the evaluation of their interactions with the permeability barrier.^5^ Also, by changing the lipid composition of SCLLs, different pathological conditions can be modelled, *e.g.* the accumulation of cholesterol sulfate and decreased cholesterol levels are specific for ichthyosis patients.^6^

To overcome the barrier function of the SC, chemical penetration enhancers (CPEs) are conventional and effective approaches as they reduce skin barrier resistance and thus promote the penetration of drugs.^2,6^ The mechanism of action of CPEs is thought to be by disrupting the packing of skin lipids and altering the barrier of the SC, by changing the partitioning behaviour of the drug at the SC-viable epidermis interface or by affecting the thermodynamic activity of the drug.^7,8^ The evaluation of the exact mechanism of action is crucial in the development of transdermal and dermal formulations. Conventional methods to investigate the effects of CPEs are based on the determination of the cumulative amount of skin permeation, flux, and permeability coefficient of drugs in the presence and absence of CPEs by pre-treatment and partitioning studies, spectroscopic investigations and thermal analysis.^8^ However, these approaches are usually time-consuming, complicated, and usually need skin from human or animal source.^2^ Nowadays there is an increasing awareness of animal welfare issues and there is a lack of supply of suitable human skin, which gives rise to a need to develop effective replacement approaches to avoid the use of excised skin.^8^ These intentions also support the reason for using SCLL as skin modelling systems.

There are several methods to investigate the effects of CPEs on SCLLs. Kim *et al.* employed SCLLs first to investigate the mechanism of action of CPEs using differential scanning calorimetry (DSC).^8,9^ The incorporation of fluorescent markers in the liposomes, and the study of their release is also a well-known technique to measure the stability of the vesicular membrane of SCLL, and to investigate the effects of CPEs on this stability.^10–12^

In this study, we aimed to investigate the effects of two modern penetration enhancers: Kolliphor RH40 and Transcutol.

Kolliphor RH40 (Polyoxyl 40 Hydrogenated Castor Oil USP/NF) is a nonionic surfactant reported to shift the drug distribution to the stratum corneum, but the exact mechanism was not described yet.^13,14^ Generally, non-ionic surfactants have low toxicity, however, they have also a minor enhancement effect compared to ionic types, which could be explained by the low degree of SC structure disordering effect.^15^ Breuer suggested two possible mechanisms to describe how nonionic surfactants enhance the rate of transport: (I) surfactants may penetrate into the intercellular regions of the stratum corneum, increase fluidity and, at last, solubilise and extract lipid components; (II) the penetration of a surfactant into the intercellular matrix followed by interactions and binding with keratin filaments may result in a disruption within the corneocyte.^16^

Transcutol, a monoethyl ether of diethylene glycol, is classified into the group of alcohols and glycols, which are reported to interact with the aqueous domain of the lipid bilayers, which increase the solubility of drugs in the skin.^1^ Harrison *et al.* reported that the penetration enhancement property of Transcutol is the result of changes in solubility rather than diffusion in the membrane. Another study stated that the enhancing ability of Transcutol is attributed to its ability to pass through the skin and get incorporated into the multiple lipid bilayers, thereby swelling the intercellular lipids.^13^ These results suggest that further mechanistic studies are required to elucidate its exact interaction with skin components.

The present study attempts to investigate the lipid interactions and enhancer effects of the above-mentioned CPEs in an isolated lipoidal system of SCLLs using fluorescent dye efflux and *ex vivo* penetration experiments. The correlation between novel *in vitro* SCLL leakage results and conventional *ex vivo* skin penetration results was investigated to determine the applicability of SCLL liposomes as skin modelling systems.

## Experimental

### Materials

Ceramide (type III and VI) was kindly supplied by Evonik Industries (Evonik Nutrition & Care GmbH, Essen, Germany). Cholesterol and caffeine were purchased from Hungaropharma Plc. (Budapest, Hungary). Cholesterol sulfate and EDTA were obtained from Sigma-Aldrich (Saint Louis, Missouri, USA). Palmitic acid was supplied by Mosselman s.a. (Ghlin, Belgium). Kolliphor RH40 was obtained from BASF SE (Ludwigshafen, Germany). Transcutol was a kind gift of Azelis Hungary Ltd. (Budapest, Hungary). Organic solvents (chloroform and methanol), 4-(2-hydroxyethyl)-1-piperazineethanesulfonic acid (HEPES) and sodium chloride (purity ≥ 99.5%) were from Carl Roth GmbH & Co. KG (Karlsruhe, Germany). HPTLC plates (20 × 10 cm silica-gel 60 WRF254s) were delivered by Merck (Darmstadt, Germany). Mobile-phase components, solvents for lipids and *o*-phosphoric acid 85% were purchased from Carl Roth GmbH (Karlsruhe, Germany) and were of HPLC or p.a. quality. HPLC grade water was prepared from Milli-Q water purification system (Merck Type 1, Millipore, Milford, MA, USA) and methanol (gradient grade, suitable for HPLC/UHPLC measurements) was purchased from VWR Scientific (Seattle, WA, USA).

### SCLL preparation

The preparation of SCLL liposomes followed the method described by Wertz *et al.*^5^ Briefly, individual lipids were dissolved in chloroform–methanol (2 : 1 by volume) and appropriate volumes were combined to obtain the corresponding mixture (ceramides : cholesterol : palmitic acid : cholesterol sulfate = 4 : 2.5 : 2.5 : 1, weight percent). The lipid mixture was then placed in a culture tube, and the solvent was removed with a stream of nitrogen (Christ RVC 2-18 rotary evaporator) and then under high vacuum at room temperature. Aqueous dispersions of each lipid mixture were prepared by suspension in buffer containing 20 mM HEPES, 120 mM NaCl, 0.8 mM EDTA (supplemented with 70 mM calcein dye for efflux measurements), to provide a final concentration of 5 mg mL^−1^ lipid at pH 8. The lipids were left to hydrate for 30 min with occasional shaking. The suspensions were then sonicated in a bath sonicator at 80 °C (temperatures slightly higher than those corresponding to the phase transition temperature for each lipid mixture) for about 1 h until the suspensions became clear. The preparations were then annealed at the same temperature for 30 min. Liposomal formulations were stored at 4 °C and used within 1 day.

### Particle size and polydispersity index of SCLL

The mean vesicle size and polydispersity indexes of liposome suspensions were determined using Malvern Nano ZS system (Malvern Instruments, Malvern, UK) based on dynamic light scattering. The samples were diluted with their aqueous phase in order to avoid multiscattering phenomena, and then they were incubated at 25 °C. Measurements were made in standard disposable cuvettes, each of them in triplicate. The polydispersity index was also calculated to evaluate the homogeneity of the dispersion.

### Calcein efflux and lifetime-based fluorescence leakage assay

Calcein leakage assay is based on the fact that the entrapped calcein expresses only slight fluorescence due to self-quenching or co-entrapment of a quencher, but when it is released from the liposomes and attenuates, it shows fluorescence. Efflux *E* is defined as the fraction of previously entrapped calcein that was released within a defined time point after exposure to the permeabilizer.^17^ The degree of calcein efflux from SCLLs refers to the degree of disruption effect brought about by CPEs.

The prompt-deconvoluted fit of the decay curve after freeing all calcein in a vesicle dispersion provides a virtually monoexponential behavior with a lifetime of *τ* = 4.0 ns. The curve for calcein-loaded vesicles in the absence of a permeabilizer results in a biexponential behavior with a dominant *B*_E_ (subscript E for entrapped) with *τ* = 0.4 ns and a weak *B*_F_0__ (subscript F for free and 0 for ‘without permeabilizer’) with *τ* = 4.0 ns from some remaining free dye. Generally, increasing detergent concentrations lead to a growth of the long-lived component (free dye) at the expense of the short-lived one (entrapped). This can be quantified as the efflux.

Calcein efflux (*E*) was calculated according to the following equation:^18^1
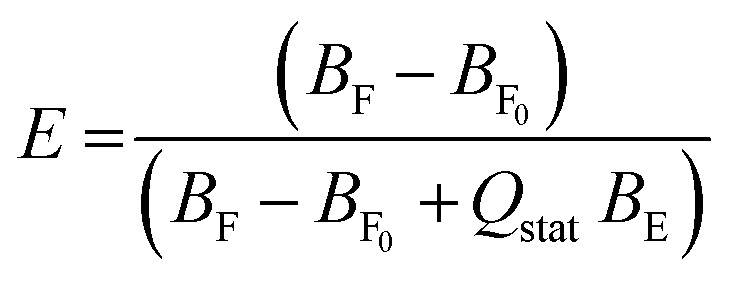
where *B* is the pre-exponential factor (proportional to the concentrations of free and entrapped dye). *B*_F_0__ corresponds to the free dye at the beginning of the measurement (this corrects for incomplete dye removal on the column), *B*_F_ to the free dye concentration at certain time points and *B*_E_ to the entrapped calcein concentration. *Q*_stat_ is the static quenching factor, which considers that some entrapped dye might be ‘invisible’ due to dimerization in the ground state. We used 1.2 as *Q*_stat_ based on the literature.^18^

Before the fluorescence measurements, vesicles were separated from unencapsulated fluorescent dye on a PD-10 de-salting column (GE Healthcare) using HEPES buffer (20 mM HEPES, 120 mM NaCl, 0.8 mM EDTA) as the mobile phase. 5 μL aliquots of these vesicles were incubated with different concentrations of surfactant, at room temperature on a gently rotating shaker and efflux measurements were performed after 1, 10, 30 min, 1, 2, 3 h. The samples were placed in a Horiba Jobin Yvon (Edison, NJ) Fluorolog 3 system equipped with a 467 nm laser diode pulsed at 1 MHz for excitation; the decay curve was recorded at a wavelength of 515 nm (bandwidth 2 nm) for 25 s using time-correlated single photon counting (TCSPC). We used HORIBA DAS6 software to fit decay curves biexponentially by deconvoluting them with the instrument response function as measured with a scattering LUDOX solution.

### *Ex vivo* skin penetration experiments

For the penetration experiments, heat separated human epidermis was used.^19^ Excised human skin was obtained from a Caucasian female patient. All experiments were performed in accordance with the OECD Guideline 428: Skin Absorption: *In Vitro* Method (2004). Experiments were approved by the Ethical Committee of University of Szeged (Albert Szent-Györgyi Clinical Centre, Human Investigation Review Board license number: 83/2008.). Informed consents were obtained from human participants of this study. The separated epidermal membrane was applied onto the surface of PBS (phosphate buffer solution, pH = 7.4) for at least 20 min, then set on a supporting mixed cellulose ester membrane (Porafil, Machenerey-Nagel, Düren, Germany; pore diameter 0.45 μm) and mounted in a vertical Franz diffusion cell (Hanson Research, Chatsworth, CA, USA) in a six-unit assembly (effective permeation area 1.676 cm^2^) over 24 h at 32 ± 0.5 °C. PBS pH 7.4 was used as the acceptor medium and it was stirred at 450 rpm throughout the experiment. At selected time intervals, samples of 0.8 mL were taken from the acceptor phase by the autosampler (Hanson Research, Chatsworth, CA, USA) and replaced with an equal volume of fresh receiver medium.

The donor chamber was filled with 1 mL of 2 mg mL^−1^ caffeine solution, providing infinite dosing, with defined amounts of CPEs in PBS. The amount of permeated drug was measured using a high-performance liquid chromatography (HPLC) system.

Five parallel measurements were carried out and the amount of caffeine penetrating during a time period was plotted. The results were expressed as means ± SD for the evaluation of drug release kinetics.

The penetration of caffeine was calculated in terms of the mean cumulative amount permeated through the membrane, taking the diffusion area into account. The results were plotted as a function of time.

### HPLC analysis of caffeine

The HPLC analysis of caffeine was performed with a Shimadzu Nexera X2 UHPLC system. The system control and data acquisition were performed with Shimadzu LabSolutions software package. The chromatographic separation was achieved by Phenomenex Kinetex C18 column with 2.6 μm particle size (150 × 4.6 mm I.D.). The column temperature was maintained at 25 °C. The separations were carried out in isocratic mode. The mobile phase of pure HPLC grade water and methanol in the ratio of 75 : 25 was pumped at a flow rate of 1.0 mL min^−1^. For the detection of caffeine, a PDA detector was used. The wavelength of detection was 272 nm and the detector cell temperature was adjusted to 40 °C. The sample tray holder's temperature was 25 °C, the injected volume of the samples was 5 μL. The analysis time was 7 min, the retention time of caffeine was ∼5.1 min.

## Results

### SCLL characteristics: particle size and polydispersity index

**Fig. 1:**
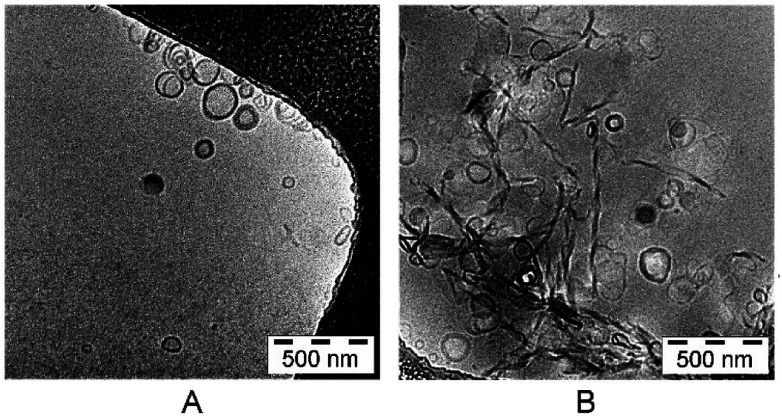
Transmission electron microscope images of SCLLs (A) and calcein-loaded SCLLs (B).

The lipid mixture, representing stratum corneum lipids, formed liposomes in the size of 135.23 ± 0.06 nm. The polydispersity index (PDI) was 0.220 ± 0.02 (Table 1). The particle size of the calcein-loaded SCLL was 137.35 ± 0.07 nm and their polydispersity index was 0.228 ± 0.02. The electron microscopic images revealed that most of the particles were unilamellar (Fig. 1).

**Table 1:** Particle size and PDI of SCLLs with and without calcein

	*Z*-ave (nm)	PDI
SCLL	135.23 ± 0.06	0.220 ± 0.02
Calcein-loaded SCLL	137.35 ± 0.07	0.228 ± 0.02

### Calcein efflux and lifetime-based fluorescence leakage assay

The effect of different concentrations of Kolliphor RH40 and Transcutol was tested on the disruption of SCLLs applying calcein leakage assay.

**Fig. 2:**
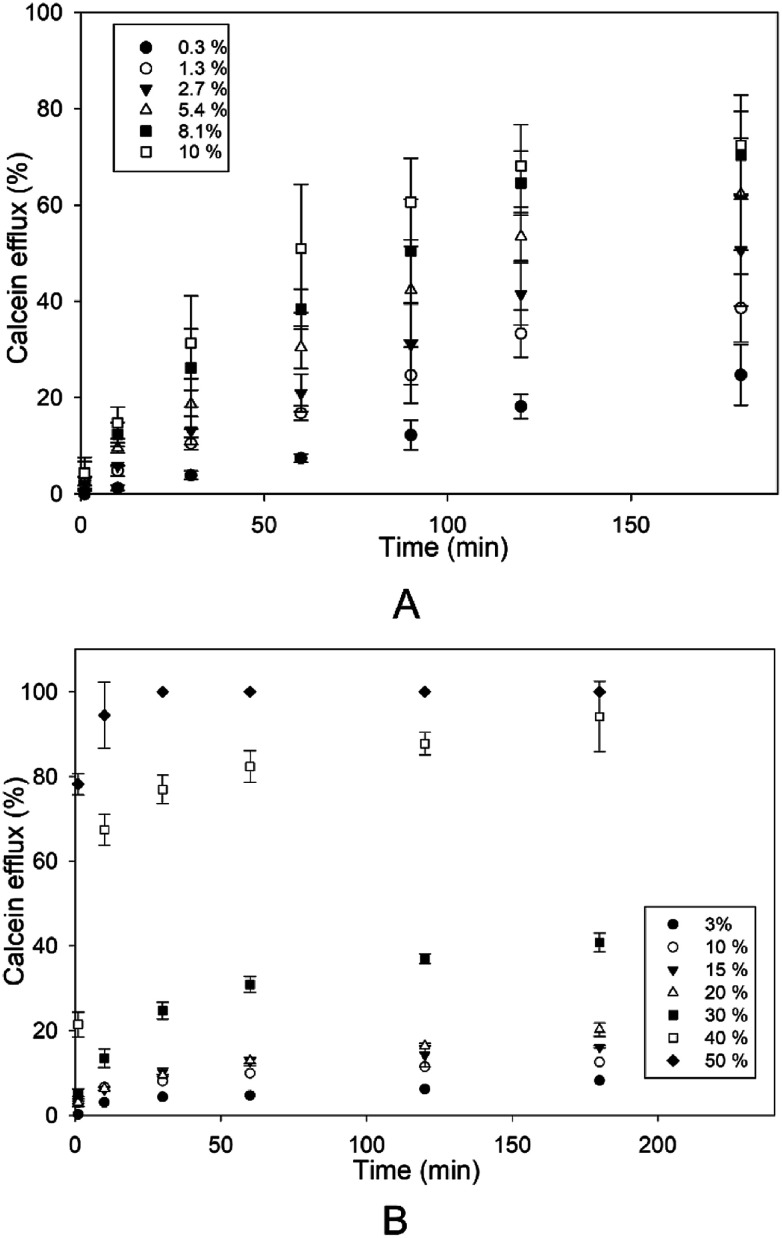
Efflux from SCLLs induced by Kolliphor RH40 (A) and Transcutol (B) as a function of incubation time. The concentrations of the CPEs are indicated in the plot.

First, the correlation between the enhancer concentration and the efflux was investigated for 3 hours (Fig. 2). Both Kolliphor RH40 and Transcutol promoted calcein efflux from SCLLs in a concentration dependent manner. However, from Transcutol, much higher concentration was needed to achieve the same efflux value. The efflux curve for Transcutol reaches a plateau value after about 1 hour, while the calcein efflux caused by Kolliphor RH40 increases even after 2 hours. For longer waiting times, there is only a slight further increase with a much slower rate.

**Fig. 3:**
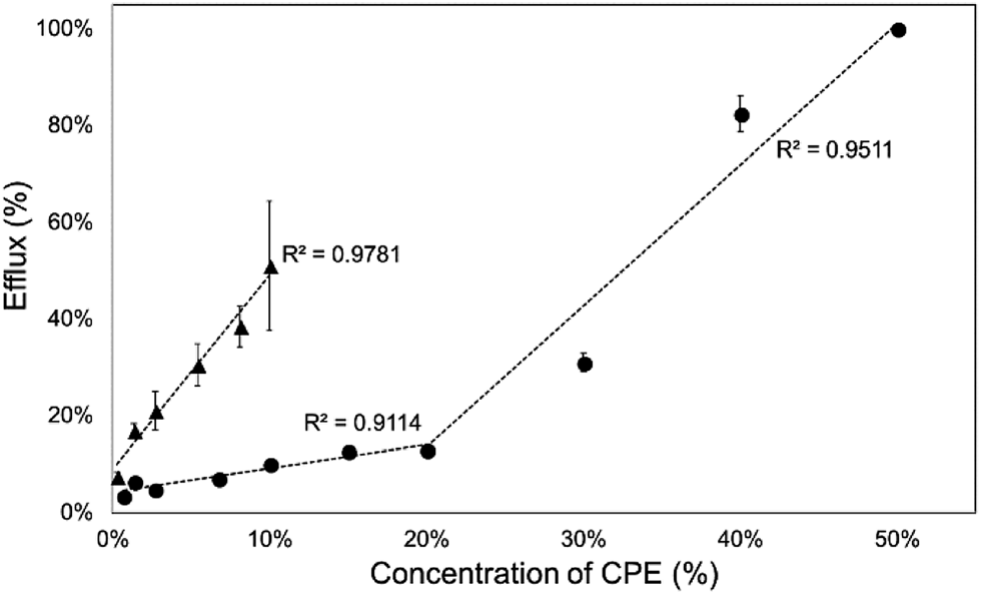
Calcein efflux from SCLLs after 1 h of incubation as a function of CPE concentration (▲: Kolliphor RH40, ●: Transcutol).

Then, we investigated the relationship between CPE concentration and calcein efflux after 1 hour. The concentration profiles of the efflux were different for the two investigated CPEs. Kolliphor RH40 showed a linear relationship between calcein efflux and concentration, while in case of Transcutol a concave downward profile was observable, which indicated the limit level for the disruption of SCLLs (Fig. 3).

However, these results do not give detailed information about the characteristics of vesicle disruption. The efflux can result mainly from two mechanisms: (i) all-or-none, when only some vesicles are disrupted (and releasing all entrapped dye) while others remain undamaged or (ii) graded, when all vesicles release a certain fraction of their content.

The fluorescence lifetime measurement method published by Patel *et al.*^18^ can distinguish between these two mechanisms by plotting calcein efflux (*E*) *versus* lifetime of entrapped dye (*τ*_E_). Ideally, homogeneously graded leakage points of the above-mentioned graph should lie on the diagonal (efflux *E* couples directly with the entrapped calcein concentration: the more has leaked out, the less is left behind, which correlates with *τ*_E_). The right-shift of *E*(*τ*_E_) from the diagonal is a measure for the heterogeneity of graded leakage. All-or-none leakage increases *E*, but does not affect *τ*_E_ since the entrapped calcein concentration in the non-leaky vesicles remains 0.4 ns and the empty vesicles do not contribute to *τ*_E_ at all, thus *E*(*τ*_E_) should follow a vertical line at *τ*_E_ 0.4.

**Fig. 4:**
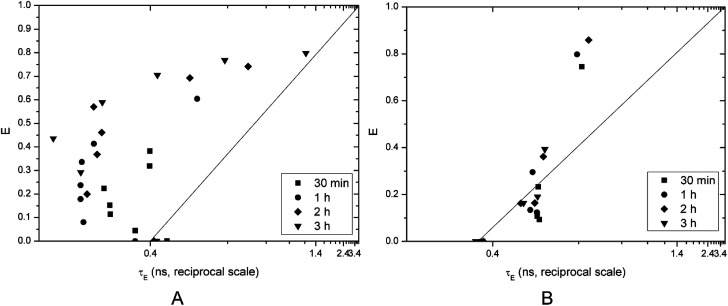
Calcein efflux as a function of the entrapped fluorescence lifetime on a reciprocal scale brought on by Kolliphor RH40 (A) and Transcutol (B).

Vesicles incubated with Kolliphor RH40 showed all-or-none leakage mechanism until a certain point around 60% efflux, then it became graded. The efflux increases with increasing concentration of Kolliphor RH40 and with time (Fig. 4A).

Transcutol caused graded leakage of the vesicles, which is in agreement with the previous results: calcein efflux is not promoted at lower concentrations, a certain concentration of Transcutol is needed to increase the efflux to a great extent (Fig. 4B). This finding is in agreement with a previous study.^1^ Transcutol is reported to interact in the aqueous domain of the lipid bilayers which increase the solubility of drugs in the skin. According to Moghadam *et al.*, Transcutol caused only a slight disordering effect within the SC membrane. They also proved the superior enhancer effect of Kolliphor RH40.^14^

### Effect of CPEs on *ex vivo* penetration of caffeine

**Fig. 5:**
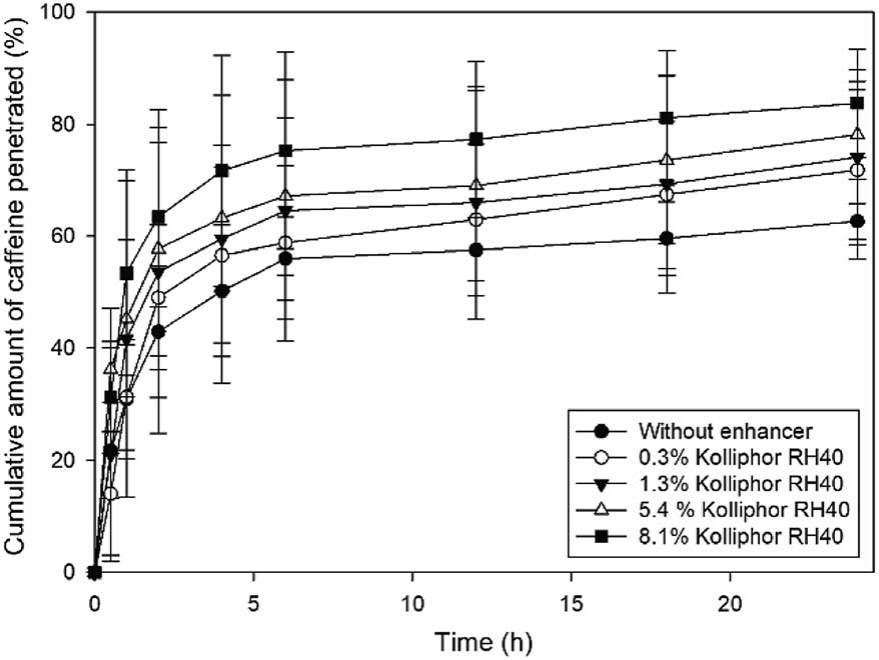
Cumulative amount of caffeine penetrated through heat separated epidermis after application of different concentrations of Kolliphor RH40.

In this work, we selected caffeine as a hydrophilic model drug to determine the penetration enhancer effect of different concentrations of Kolliphor RH40 on human epidermis *ex vivo*. Fig. 5 shows the cumulative amounts of caffeine penetrated through heat separated epidermis over 24 hours.

It can be stated that the *ex vivo* penetration of caffeine was strongly dependent on the concentration of Kolliphor RH40. With an increasing concentration of the penetration enhancer, the skin penetration of caffeine also increased. The highest concentration of Kolliphor RH40 applied was 8.1% and it caused 21% increase in the penetration of caffeine compared to the blank caffeine solution after 24 h.

### Relationship between calcein leakage and *ex vivo* skin penetration

Calcein leakage assay performed with SCLLs was applied as an *in vitro* model to investigate the enhancing effect of CPEs. We attempted to evaluate the correlation between the *in vitro* SCLL-based data and the *ex vivo* results gained by conventional penetration experiments. For this reason, we evaluated the change in the penetrated amount of caffeine compared to the blank formulation and the increase in efflux *in vitro* compared to SCLLs without enhancer after 1 and 2 hours.

**Fig. 6:**
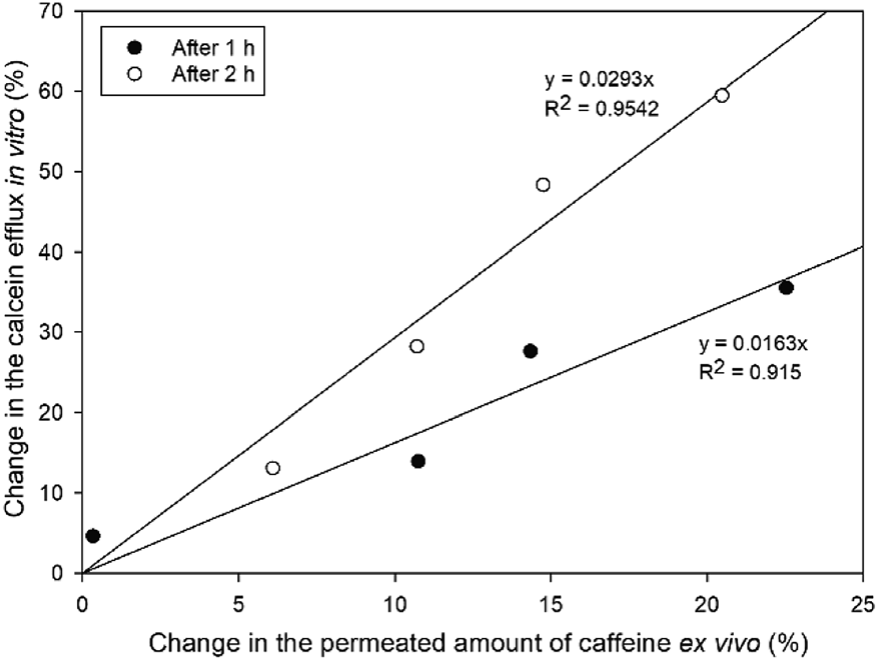
Relationship between calcein leakage and increase in the caffeine permeated compared to blank formulation after 1 and 2 hours.

Fig. 6 shows the relationship between the increase in the cumulative permeated amount of caffeine compared to blank solution and calcein leakage from SCLLs. The correlation coefficient of this relationship was 0.915 after 1 h and 0.954 after 2 h. According to the results, Kolliphor RH40 affected SCLL disruption more markedly than the skin permeation of caffeine, which could be the result of different lipid-enhancer ratios in these cases.

## Conclusion

Most chemical penetration enhancers act upon the skin by permeabilizing the lipid bilayer membranes in the stratum corneum. The permeabilizing mechanism can be assessed by vesicle leakage experiments that use model membranes, with the assumption that biological activity arises from permeabilization of the lipid bilayer. To this end, we used a lifetime-based leakage assay with calcein-loaded vesicles to study and compare the membrane permeabilizing properties of different concentrations of novel CPEs like Kolliphor RH40 and Transcutol.

We attempted to compare the results of this *in vitro* high throughput screening method with conventional *ex vivo* penetration study results in case of Kolliphor RH40. Both the efflux *in vitro* and caffeine penetration *ex vivo* were concentration-dependent.

Our results suggest that SCLLs could be promising *in vitro* approaches for screening the effects and effective concentrations of chemical penetration enhancers as we found a good correlation between SCLL-based experiments and the skin penetration study.

## Conflicts of interest

There are no conflicts to declare.

## Supplementary Material
